# Phenotypic Screening of Prospective Analgesics Among FDA‐Approved Compounds using an iPSC‐Based Model of Acute and Chronic Inflammatory Nociception

**DOI:** 10.1002/advs.202303724

**Published:** 2024-01-08

**Authors:** Bryan James Black, Rasha El Ghazal, Neal Lojek, Victoria Williams, Jai Singh Rajput, Jennifer M. Lawson

**Affiliations:** ^1^ Department of Biomedical Engineering Francis College of Engineering University of Massachusetts Lowell Lowell MA 01854 USA

**Keywords:** drug screening, hiPSC tissue model, inflammatory nociception, microelectrode arrays, phenotypic screening

## Abstract

Classical target‐based drug screening is low‐throughput, largely subjective, and costly. Phenotypic screening based on in vitro models is increasingly being used to identify candidate compounds that modulate complex cell/tissue functions. Chronic inflammatory nociception, and subsequent chronic pain conditions, affect peripheral sensory neuron activity (e.g., firing of action potentials) through myriad pathways, and remain unaddressed in regard to effective, non‐addictive management/treatment options. Here, a chronic inflammatory nociception model is demonstrated based on induced pluripotent stem cell (iPSC) sensory neurons and glia, co‐cultured on microelectrode arrays (MEAs). iPSC sensory co‐cultures exhibit coordinated spontaneous extracellular action potential (EAP) firing, reaching a stable baseline after ≈27 days in vitro (DIV). Spontaneous and evoked EAP metrics are significantly modulated by 24‐h incubation with tumor necrosis factor‐alpha (TNF‐α), representing an inflammatory phenotype. Compared with positive controls (lidocaine), this model is identified as an “excellent” stand‐alone assay based on a modified Z’ assay quality metric. This model is then used to screen 15 cherry‐picked, off‐label, Food and Drug Administration (FDA)‐approved compounds; 10 of 15 are identified as “hits”. Both hits and “misses” are discussed in turn. In total, this data suggests that iPSC sensory co‐cultures on MEAs may represent a moderate‐to‐high‐throughput assay for drug discovery targeting inflammatory nociception.

## Graphical abstract

Chronic pain remains the number one public health crisis in the US and around the world. Phenotypic screening assays for identifying novel analgesics are desperately needed. Here, the authors report on the development and characterization of an inflammatory nociception screening assay based on spontaneous and evoked activity from human induced pluripotent sensory neurons and glial cells co‐cultured on microelectrode arrays.

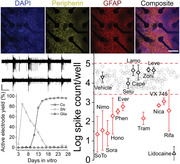

## 1 Introduction

Nociception is the process by which noxious stimuli are detected and transmitted from the peripheral to the central nervous system. Nociception and its perception as pain are necessary for survival, but pathological states affecting nociceptors may result in chronic pain conditions due to maladaptive changes in the peripheral and/or central nervous systems. Chronic pain is defined as pain that persists after the apparent underlying cause (e.g., disease or injury) has resolved. This broadly defined pathology is estimated to affect one‐sixth of the global population^[^ ^1^, ^2^, ^3^, ^4^ ^]^ and has been linked to disability, drug or alcohol dependency, reduced quality of life, anxiety, and depression.^[^ ^5^ ^]^ Existing pain management options based on pharmaceutical drugs (i.e., opioids) lead to tolerance, dependency, abuse, and reduced quality of life.^[^ ^5^, ^6^ ^]^ Current analgesic screening methodologies based on the identification and exploitation of “druggable” targets are low‐throughput and ineffective, and current preclinical in vitro/vivo models and methods for identifying novel analgesics are problematic.^[^ ^7^ ^]^ Rodent behavioral models are widely used and genetically modified strains can be advantageous for mechanistic studies. However, animal behavioral studies targeting analgesic “hit” or “lead” discovery are expensive, rely on primarily subjective measurements, and are relatively low‐throughput.^[^ ^8^ ^]^ Importantly, studies have shown that rodent nociceptors (sensory neurons responsible for the detection of noxious stimuli) exhibit important proteomic, genomic, and phenotypic differences when compared to their human counterparts.^[^ ^9^ ^]^ Furthermore, animal species‐to‐species variability leads to preclinical false positives or false negatives, potentially leading to clinical trial failure.^[^ ^10^, ^11^ ^]^ Immortalized cell lines, which may be transfected to express pain‐relevant recombinant proteins such as transient receptor potential vanilloid 1 (TRPV1)^[^ ^12^ ^]^ are likewise inadequate in their recapitulation of certain complex pathological phenotypes,^[^ ^13^ ^]^ inadequate in their subgroup representation or classification, or, in some cases, even lack expression of presumptive nociceptive markers.^[^ ^14^ ^]^ The use of primary, cadaverous human tissues overcomes some of these challenges, yet it is highly limited by tissue availability – both in terms of the number of donors and number of extractable dorsal root ganglia (DRG) – as well as viability and epi‐/genetic variance among donor populations.^[^ ^15^ ^]^ However, human induced pluripotent stem cells (hiPSCs) seem to^[^ ^16^, ^17^, ^18^, ^19^, ^20^ ^]^ and may be integrated with moderate‐to‐high throughput phenotypic screening platforms,^[^ ^21^ ^]^ such as those used in high‐throughput functional imaging^[^ ^22^ ^]^ as well as multi‐well microelectrode arrays (MEAs).^[^ ^23^ ^]^ Multi‐well MEAs record high‐content phenotypic information with excellent signal‐to‐noise ratios (SNR) in a long‐term, repeatable, non‐invasive way. While hiPSC‐derived sensory neurons and/or nociceptors mono‐cultured on MEAs have been used for validation of single, patient‐specific compounds,^[^ ^24^ ^]^ no published study has validated sensory co‐cultures as a viable method for moderate‐to‐high throughput “hit” or “lead” detection using a broad range of candidate molecules.

Here, we report the development of an acute and chronic nociception model based on hiPSC nociceptors and glial cells cultured on multi‐well MEAs for the purposes of novel antinociceptive “hit” or “lead” discovery. In total, we demonstrate 1) the ability to differentiate and retain multiple hiPSC cell types in co‐culture, 2) stable spontaneous and evoked activity, including extracellular action potentials (EAPs) and oscillating calcium transience (OCaTs), recorded from MEAs over at least 45 days in vitro (DIV), 3) sensory co‐culture sensitivity to inflammatory cytokine tumor necrosis factor‐alpha (TNF‐α), 4) that these protocols result in an excellent assay based on modified assay quality metrics (Z’), and 5) feasibility as a screening methodology based on screening cherry‐picked Food and Drug Administration (FDA)‐approved off‐label compounds.

## 2 Results

### 2.1 hiPSC Sensory Co‐Cultures are Comprised of Sensory Neurons and Glial Cells

**Figure 1:**
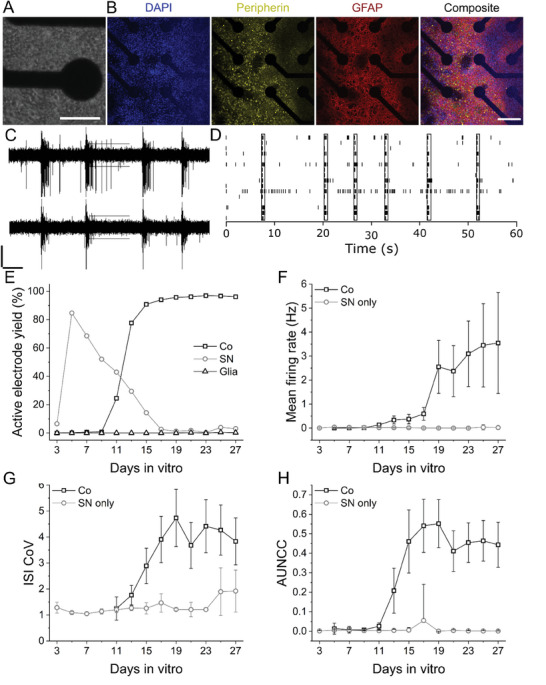
Co‐cultures of hiPSC sensory neurons and glia exhibit spontaneous, time‐progressing EAP firing. A) Representative phase image of hiPSC co‐cultures on MEAs. Scale bar 100 µm. B) ICC staining of co‐cultures for nuclear marker DAPI (blue), astrocyte marker GFAP (red), and peripheral neuronal marker peripherin (PRPH, yellow). Scale bar 250 µm C) Representative filtered traces of voltage versus time for two electrodes in a single well co‐culture. Vertical and horizontal scale bars represent 8 µV and 2 s, respectively. D) Raster plot of 5.5 σ threshold crossings across a single well co‐culture exhibiting network level activity (black rectangular outlines). E) Time progression of cumulative active electrode yield % and mean ± SD of MFR, ISI CoV, and AUNCC through DIV27 for hiPSC sensory mono‐ and co‐cultures.

To determine whether our culture protocols resulted in the differentiation of distinguishable sensory neurons and glia, matured sensory co‐cultures were immunolabeled using canonical markers for neuron (neuronal nuclei, NeuN), sensory neurons (peripherin, PRHP), and glia (glial fibrillary acidic protein, GFAP). **Figure** **1** shows both phase contrast (Figure 1A) and confocal images (Figure 1B) of sensory co‐cultures on MEAs. ICC images indicate a high density of PRHP+ neurons (913 ± 169 cells mm^−2^) within 100 µm of electrodes as well as a clear distinction between GFAP+ glia and PRHP+ sensory neurons.

### 2.2 hiPSC Sensory Neuron and Glia Co‐Cultures Exhibit Stable Spontaneous EAP Activity

To determine whether sensory progenitors (Anatomic, Inc.) developed into more relevant and robust models of acute and chronic nociception in co‐culture, we performed spontaneous and evoked EAP recordings using multi‐well MEAs. with three culture groups; sensory neurons (SN) only, spinal astrocytes (glia) only, or sensory co‐culture (30k SN and 20k glia per well). Figure 1C shows representative continuous traces, sampled at 12.5 kHz from 768 electrodes separated into 48 wells simultaneously. Synchronous firing across 16 electrodes from a representative well is shown in Figure 1D. Mono‐cultures of spinal glia did not exhibit a single spontaneously active electrode throughout the lifetime of the culture (at least 28 days), as expected (Figure 1E). SN mono‐cultures exhibited active electrodes, reaching an active electrode yield percentage (AEY%) of 84.6% by DIV 5, but subsequently decreasing to 1.3% by DIV 19. Mono‐cultures also exhibited increasing mean firing rate (MFR) through DIV 4, reaching a maximum of 0.038 ± 0.014 Hz. However, MFR decreased to 0.001 ± 0.004 Hz by DIV 18. Sensory co‐cultures AEY% reached a maximum of 96.6% by DIV 23. Additionally, 47 of 48 wells were classified as “active” (greater than four active electrodes per well) based on previous statistical cases made for high‐throughput MEA requirements.^[^ ^25^ ^]^ Spontaneous EAP MFR for co‐cultures increased from 0.004 ± 0.001 Hz on DIV 7 to 3.55 ± 2.10 Hz on DIV 27. Importantly, sensory co‐cultures reached stable spontaneous activity by DIV 27 as defined by statistically similar AEY% and weighted mean firing rate (WMFR) across three consecutive recordings based on a two‐sample test of proportions and Kruskal‐Wallis ANOVA (KWANOVA), respectively (Figure 1E,F). Additionally, we observed marked differences in inter‐spike interval coefficient of variance (ISI CoV) and area under the normalized cross‐correlogram (AUNCC) between co‐cultures and monocultures after DIV 13, with co‐cultures showing substantially higher values for both measures (Figure 1G,H, 3.83 ± 0.90 vs 1.92 ± 0.81 and 0.44 ± 0.12 vs 8.78E‐4 ± 7.60E‐4, *p* = 5.02E‐16 and 6.46E‐7, respectively) on DIV 27.

### 2.3 Spontaneous Network‐Level Activity is Mediated by Glutamatergic Synapses

**Figure 2:**
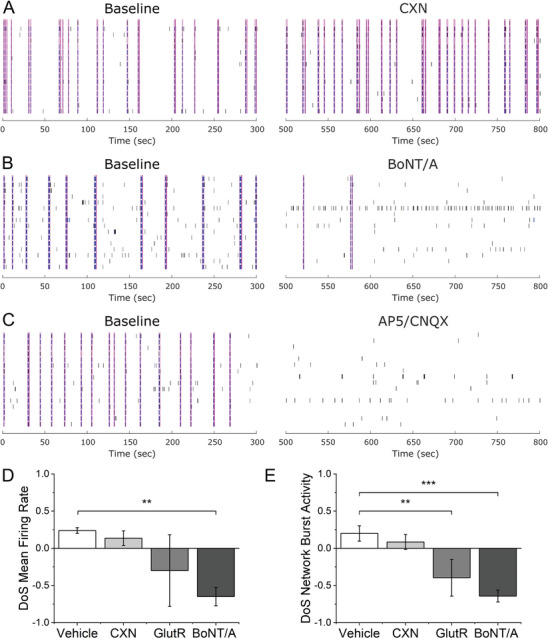
Spontaneous network activity is mediated by glutamatergic synapses. A–C) Representative raster plots before (Baseline) and after the addition of carbenoxolone (CXN), botulinum neurotoxin serotype A (BoNT/A), and a combination of APV/CNQX (GlutR). Pink vertical bars represent network bursts. Blue blocks represent electrode‐level bursts. D,E) Quantification of difference‐over‐sum‐normalized changes in D) mean firing rate and E) network burst rate. *, **, *** represent *p* < 0.05, 0.01, and 0.001, respectively.

To determine whether our observations of coordinated, “synchronous” activity across electrodes is mediated by gap junction communication or functional, synaptic networks, we separately recorded spontaneous EAP activity from mature sensory co‐cultures in the presence of gap junction inhibitor carbenoxolone (CXN) or Botulinum neurotoxin serotype A (BoNT/A). **Figure** **2**A shows spontaneous raster plots before and after 50 µM CXN. CXN treatment reduced MFR by 22.5% (1.24 ± 0.24 vs 1.61 ± 0.62 Hz) within 15 min but this reduction was not statistically significant. The reductions seen in the AUNCC, a synchrony metric (0.658 ± 0.038 vs 0.690 ± 0.019), and network bursting rates (0.052 ± 0.018 vs 0.063 ± 0.007 Hz) were also insignificant. BoNT/A (1 nM) significantly reduced MFR (0.11 ± 0.06 vs 0.58 ± 0.31 Hz), AUNCC (7.87E‐4 ± 5.30E‐4 vs 0.41 ± 0.08), and bursting rate (0.024 ± 0.017 vs 0.043 ± 0.006 Hz) at the 24‐h time point. To further determine whether network activity was mediated by glutamatergic synapses, we treated mature sensory co‐cultures with a combination of N‐methyl‐D‐aspartate and alpha‐amino‐3‐hydroxy‐5‐methyl‐4‐isoxazolepropionic acid receptors (NMDAR/AMPAR) antagonists ((2R)‐amino‐5‐phosphonopnetanoate/cyanquixaline, APV/CNQX). APV/CNQX (10/25 µM) significantly reduced MFR (0.029 ± 0.014 vs 2.71 ± 1.46 Hz), AUNCC (7.83E‐4 ± 5.77E‐4 vs 0.620 ± 0.081), and bursting rate (0.002 ± 9.88E‐4 vs 0.066 ± 0.016 Hz) within 15 min. Figure 2A–C shows representative raster plots for all three treatment groups. Vehicle (DI water) did not significantly affect MFR (1.52 ± 0.77 vs 1.04 ± 0.57 Hz), AUNCC (0.57 ± 0.23 vs 0.62 ± 0.25), or bursting rate (0.081 ± 0.009 vs 0.063 ± 0.010 Hz). In total, this data suggests that spontaneous activity and spontaneous network activity are predominantly due to excitatory transmission via glutamate and glutamate receptors.

### 2.4 Known Physical and Chemical Agonists of Nociceptor Activity Modulate hiPSC Sensory Co‐Culture Activity

**Figure 3:**
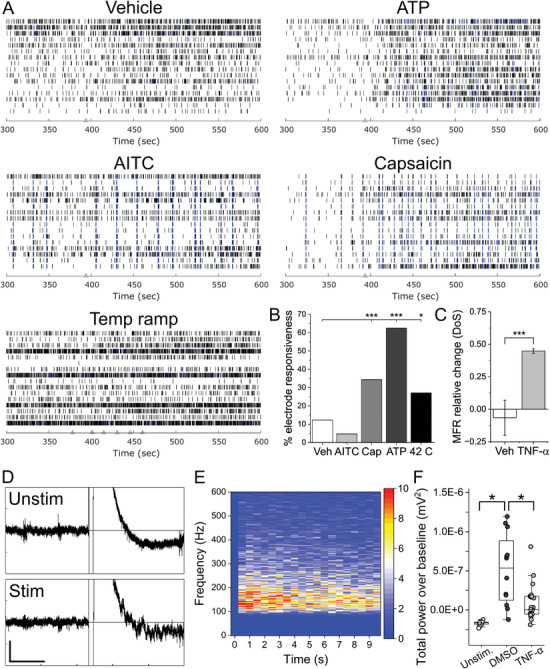
Chemical, physical, and electrical stimulation evokes activity in hiPSC sensory co‐cultures. A) Representative raster plots for vehicle, ATP, AITC, capsaicin, and temperature ramp stimulus. Grey triangles represent additional time points or degrees increase in the case of temperature ramp. B) Quantification of the percentage of electrodes exhibiting a minimum of 2‐fold increase in activity during or following stimulus (% electrode responsiveness). C) Quantification of relative change in MFR (DoS‐normalized) due to 24 h incubation with 50 ng/ml TNF‐α. *, *** indicate *p* < 0.05, 0.001, respectively. D) Representative voltage versus time traces for OCaT stimulation. Unstimulated electrodes (top) exhibit stimulus artifacts but do not exhibit OCaTs as seen on stimulated electrodes (bottom). Vertical and horizontal scale bars represent 20 µV and 1 s, respectively. E) Representative heat map of total power over baseline (TPoB) versus frequency versus time. F) OCaTs quantification for unstimulated, stimulated (without TNF‐α treatment), and stimulated following 24 h incubation with 50 ng/ml TNF‐α. * represents *p* < 0.001.

To determine whether hiPSC‐based sensory co‐cultures serve as a model of acute nociception, we exposed matured co‐cultures to capsaicin, allyl isothiocyanate (AITC), adenosine triphosphate (ATP), and a temperature ramp to 42 °C; all known chemical or physical agonists of sensory neuron subtypes. **Figure** **3**A shows representative raster plots of the EAP firing response. The percentage responsiveness was defined as the percentage of electrodes that exhibited ≥ 2‐fold increase of EAP firing rate in any 30 s bin of the treatment versus baseline recording. While AITC (50 µM) did not result in a statistically different responsiveness percentage compared to vehicle (0.1% DMSO) (12.28 and 4.69%, *p* = 0.18 based on two‐sample test of proportions, *n* = 64), capsaicin (1 µM), ATP (90 µM), and 42 °C did show statistical differences (34.38, 62.50, 27.08%, *p* = 9.70E‐4, 4.97E‐12, 0.021, respectively). Additionally, temperature ramps resulted in a statistically significant increase in network bursting rate versus baseline (0.1 ± 0.08 vs 0.21 ± 0.1 Hz). To monitor the excitability of non‐neuronal glia, OCaTs were electrically stimulated as previously described.^[^ ^26^ ^]^ Figure 3C–F shows representative traces, heat plots, and quantification of electrically‐stimulated OCaTs. Simultaneous recordings from stimulated electrodes exhibited significantly increased OCaT amplitudes while unstimulated electrodes in the same well did not show any OCaT activity (5.14E‐07 ± 4.36E‐07 vs −1.71E‐07 ± 2.86E‐08 mV^2^, *p* = 3.22E‐5, ANOVA). To determine whether incubation with inflammatory cytokine TNF‐α would elicit a hyperexcitable phenotype, we recorded spontaneous EAPs following 24 h incubation with 50 ng ml^−1^ TNF‐α. The TNF‐α‐treated group (*n* = 4 wells) exhibited statistically significant increases in relative mean firing rate (DoS‐normalized) versus vehicle (1x PBS) treatment (44.73 ± 1.79 versus −6.52 ± 13.50, *p* = 0.004).

### 2.5 Moderate Throughput, Phenotypic Screening of Cherry‐Picked, FDA‐Approved Compounds

**Figure 4:**
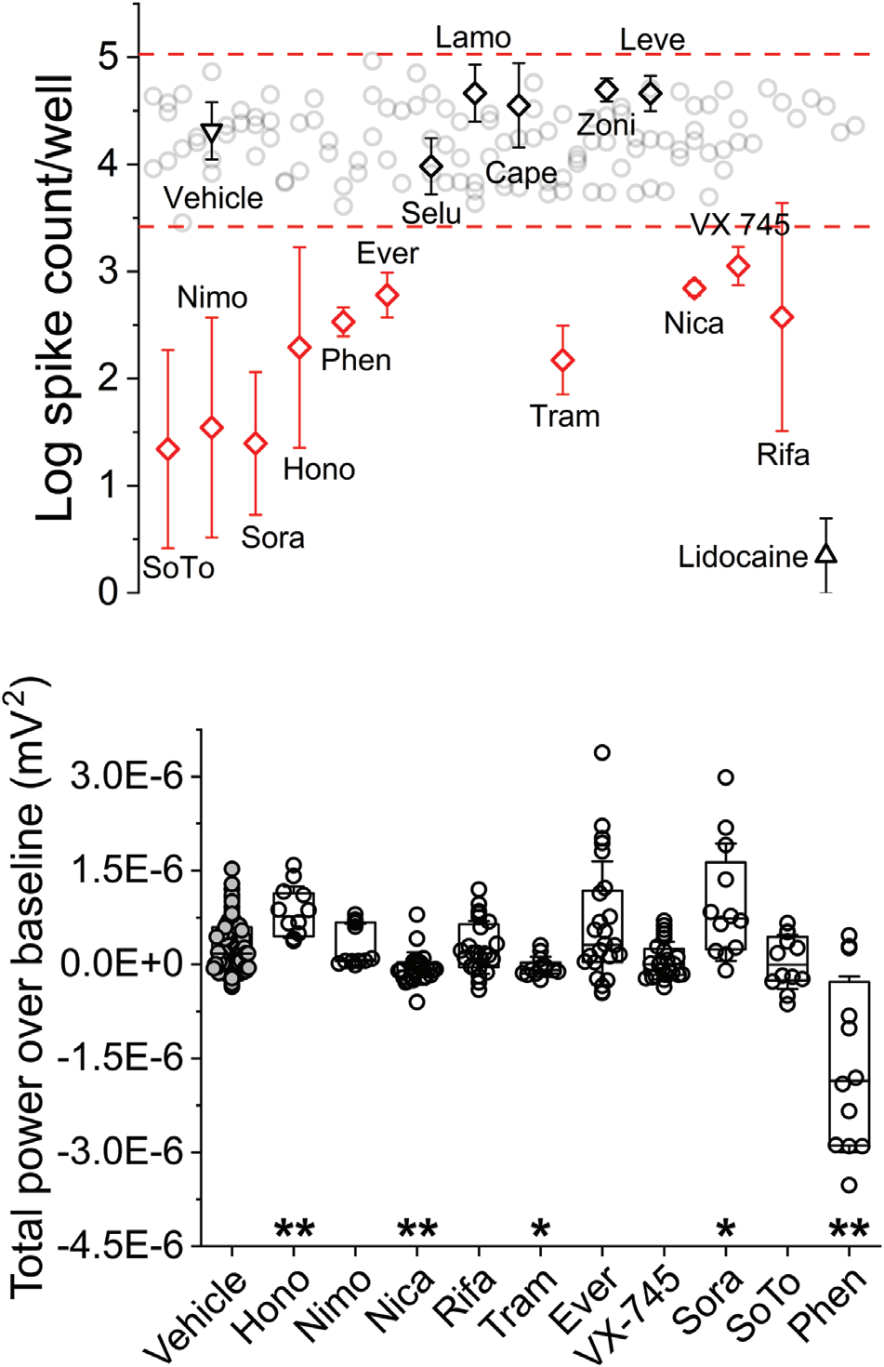
Results of EAP‐ and TPoB‐based drug screening using hiPSC sensory co‐cultures. Top) Quantification of log EAP Assessments of “hits” based on phenotypic changes after treatment (10 µM) with cherry‐picked compound. Bottom) Effect on total power over baseline (TPoB) after acute treatment on 24‐h TNF‐α incubated co‐cultures with cherry‐picked compound (* = *p* < 0.05, ** = *p* < 0.006).

To determine the quality and applicability of our assay to moderate‐throughput phenotypic screening, we first calculated a modified version of the Z’ assay quality metric, as described in^[^ ^25^ ^]^ Secondly, we selected 15 FDA‐approved compounds that were 1) CNS‐penetrant (i.e, presumed to cross the blood‐brain and blood‐nerve barriers) and 2) agonists or antagonists of pathways involved in the establishment and maintenance of chronic pain. Our Z’ calculations, using 0.1% DMSO and 100 µM lidocaine as positive and negative controls, was 0.56, indicating an excellent solo assay. For drug‐targeted pathways, we chose the MAPK signaling axis, including Raf, mTOR, MEK, and Akt, as well as sodium/calcium channels and RNA synthesis/transcription. All compounds were ordered/shipped from the same vendor (Selleck Chemicals, LLC, Houston, TX) and were provided at 10 mM stock concentration in DMSO. **Table** **1** lists compound names, generic FDA‐approved uses, and the presumed target. Drug screening was carried out across *n* = 4 wells, cumulatively, across three 48‐well plates, representing two separate cultures. **Figure** **4** (top) shows the outcome of this screening, with all compounds being added in bolus addition in DMSO to a final working concentration of 10 µM. 10 of 15 cherry‐picked compounds were registered as “hits” in addition to lidocaine, which served as our positive control. Vehicle treatment, 0.1% DMSO, resulted in 4.2 ± 0.67 log spikes versus the baseline mean of 4.1 ± 0.67 (n.s.). Interestingly, several pairs of compounds that shared similar targets fell on opposite sides of the hit line. Each compound will be addressed in turn in the Discussion section. In total, we have produced a novel, excellent‐quality, moderate‐throughput assay for screening off‐label compounds as potential analgesics.

**Table 1:** Cherry‐picked, FDA‐approved compound screening. Labeled use and target are reported as from the vendor. “Hit” indicates compounds whose average log spike count per well fell below 3× the baseline MAD. *p*‐values refer to TPoB comparisons between each compound and vehicle treatment (0.1% DMSO), based on a one‐way KWANOVA and subsequent Dunn's post‐hoc test. ↑, ↓ indicates whether TPoB was increased or decreased significantly, respectively.

Compound	Labeled use	Target	Hit/‐	TPoB [× 10^−7^ mV^2^]	*p*‐value (↑,↓)
Honokiol	Inflammation	Akt, MEK	Hit	8.33 ± 3.91	3.4E‐04 (↑)
Nimodipine	Cardiovascular disease	Calcium channel	Hit	3.14 ± 3.24	0.35
Levetiracetam	Neurological disease	Calcium channel	‐	4.15 ± 5.16	0.20
Nicardipine HCl	Cardiovascular disease	Calcium channel	Hit	−0.67 ± 2.55	2.0E‐04 (↓)
Capecitabine	Cancer	DNA/RNA synthesis	‐	3.26 ± 4.67	0.77
Rifampin	Infection	DNA/RNA synthesis	Hit	2.71 ± 4.12	0.81
Selumetinib	Cancer	MEK	‐	6.42 ± 5.29	0.02 (↑)
Trametinib	Cancer	MEK	Hit	−0.39 ± 1.54	0.02 (↓)
Everolimus	Cancer	mTOR	Hit	6.6 ± 9.63	0.17
VX‐745	Inflammation	p38 MAPK	Hit	0.61 ± 2.95	0.03
Sorafenib	Cancer	PDGFR, Raf, VEGFR	Hit	9.93 ± 8.96	0.003 (↑)
Sorafenib tosylate	Cancer	Raf	Hit	0.39 ± 4.13	0.09
Zonisamide	Cancer	Sodium channel	‐	3.05 ± 5.89	0.83
Phenytoin sodium	Neurological disease	Sodium channel	Hit	−15.92 ± 13.44	1.5E‐04 (↓)
Lamotrigine	Neurological disease	Sodium channel	‐	2.98 ± 5.63	0.68

### 2.6 Characterizing Effects of Cherry‐Picked, FDA‐Approved Compounds on OCaT Activity

To determine whether cherry‐picked compounds also modulated astrocyte activity, we calculated total power over baseline (TPoB) from electrically evoked (800 mV, 600 µs, 10 pulses, 0.025 Hz) recordings filtered from 100 Hz to 600 Hz. Table 1 also shows calculated TPoB values from stimulation trials conducted 3 h after treatment with each cherry‐picked compound.

## 3 Discussion

There is an urgent need for alternative pharmaceutical treatment options for chronic pain sufferers and a corresponding need for tissue models and assays that efficiently and effectively identify them. Here, we report on the development of a moderate throughput phenotypic assay based on hiPSC sensory co‐cultures and MEAs for identifying novel analgesics targeting inflammatory nociception. Figure 1 illustrates that distinct populations of differentiated peripheral sensory neurons (PRHP+) and glia (GFAP+) are present in culture at DIV 32. Additionally, RNAseq data confirms the presence and upregulation of sensory neuron‐associated transcripts in co‐culture conditions.^[^ ^27^ ^]^ Many previous studies have relied exclusively on either ICC or qPCR to confirm the expression of well‐established sensory neuron maturity markers, such as TRPV1, peripherin, Brn3a, and/or NTRK mRNA or proteins to denote sensory neuron maturation. In general, the interpretation of these measures (based purely on the relative abundance of sensory neuron markers) suggests phenotypic maturation of hESCs or hiPSCs into sensory neuron or nociceptive cell types. In addition to neuronal markers associated with sensory identity and maturity, several studies have confirmed and/or quantified the expression of pain‐relevant proteins such as Na_V_1.7,^[^ ^28^ ^]^ Na_V_1.8,^[^ ^29^ ^]^ ASIC,^[^ ^29^ ^]^ and P2 × 3,^[^ ^18^ ^]^ among others. However, others have reported the absence of such proteins, or the presence of ion channels not typically expressed in peripheral nerve tissues.^[^ ^29^ ^]^ Collectively, these studies suggest that, despite the expression of nociceptor biomarkers indicative of developmental maturity, current culture and/or differentiation protocols may not result in functionally (and homogeneously) mature nociceptors. For example,^[^ ^30^ ^]^ who differentiated peripheral sensory neuron‐like cells from hiPS neural crest (NC) cells, assessed responsiveness to a battery of pro‐itch histamines/cytokines as well as capsaicin via calcium imaging. They observed a subset of capsaicin‐responsive neurons but did not, in general, observe increased/decreased sensitivity to capsaicin in combination with pro/anti‐inflammatory cytokines as observed in primary mDRG.^[^ ^31^ ^]^ Importantly, we have observed in previous studies^[^ ^27^ ^]^ that co‐cultures of sensory neurons and glia offer an environment conducive to either more rapid or more complete maturation, as indicated by the presence of markers of both maturity and presumptive nociceptor identity. In total, these data serve as both a promise and a warning: that hiPSC sensory co‐cultures may be a viable model of acute and chronic nociception, and that the field should strive toward defining the functional limitations in modeling pathology and therapeutics based on hiPSCs. While recent studies have made direct comparisons to primary animal and human tissues with some promising results,^[^ ^27^ ^]^ there are still substantial limitations and fundamental neuroscience questions that may need to be addressed with further validation and appropriate caveats.

Ultimately, the value proposition of using hiPSC‐ or hESC‐based models for phenotypic screening platforms relies on the ability to quantify phenotypically (and pathologically) relevant cellular/tissue signals. In the case of peripheral sensory neurons, the primary phenotypic signal of interest is action potential firing – though there is also considerable interest in the secretion of intercellular signaling molecules and intra/intercellular calcium dynamics. After ≈30 DIV, the optimized co‐culture, including hiPSC‐derived support glia and sensory neurons, exhibits stable spontaneous and evoked activity that is modulated by known agonists/antagonists of nociceptor activity. While cell viability under baseline conditions was not explicitly tested, we observed persistent viability (based on phase imaging, ICC results, and MEA recordings) for at least two weeks following initial phenotypic stability. Importantly, we observed increased network bursts and synchrony metrics (AUNCC) after ≈11 DIV, suggesting the formation of synaptic‐mediated functional networks. This was further validated by treatments with BoNT/A and a combination of APV/CNQX, NMDA, and AMPA receptor antagonists, respectively, which confirm synapse‐mediated activity. Previous studies using dissociated DRG sensory neuron cultures, either mouse^[^ ^31^, ^32^ ^]^ or human,^[^ ^33^ ^]^ did not report prominent network activity though limited synchronous activity was reported in some cases, especially in embryonic DRG cultures.^[^ ^32^ ^]^ This may be primarily due to relatively achievable neuronal densities since primary culture yields make it difficult to reach neuronal densities reported here (913 ± 169 cells mm^−2^) and it has been shown that synaptic density (both in total and per neuron) are highly dependent on neuronal density.^[^ ^34^, ^35^ ^]^ Additionally, glial cell density and cell type (Schwann cell versus glia) are known to play differential roles in synapse formation and function.^[^ ^36^, ^37^, ^38^, ^39^ ^]^

To validate our model's usefulness as an assay, we calculated the established modified Z’ metric – a quantified measure of assay quality – as previously described.^[^ ^25^ ^]^ Using 100 µM lidocaine treatment as a negative control, modified Z’ values suggest that this model and measurements function as an “excellent” stand‐alone assay.^[^ ^40^ ^]^ While Z’ metrics were introduced for assessing high‐throughput assays (thousands to tens of thousands of measurements per day), the statistical description of the assay should only be more restrictive, not less, based on the relatively low throughput made possible here (hundreds to thousands of possible measurements per day). To further validate our assay, we recorded spontaneous and evoked activity from sensory co‐cultures treated with 1 of 15 cherry‐picked FDA‐approved compounds. These compounds were cherry‐picked based on their known mechanism of action overlapping with one or more known to be involved in inflammatory pain. Additionally, we selected compounds approved for indications other than chronic pain, that are known to cross the blood‐brain and/or blood‐nerve barriers, and that are freely soluble in DMSO. We did not consider their published EC50 prior to screening since future, blinded studies are planned to be carried out at similar initial concentrations (10 µM). Assay “hits” were defined as the mean log spike count per well greater or less than ±3× the MAD of positive control (TNF‐α) treated wells; mimicking an inflammatory condition. 10 out of 15 cherry‐picked compounds were identified as inhibitory “hits” using this definition. As detailed below, those that were not identified as hits have EC50s greater than 10 µM. An argument can be made for increasing the initial screening concentration to 100 µM despite many pre‐clinical high‐throughput screening studies being conducted at an initial concentration of 10 µM. However, it is worth noting that compounds requiring high concentrations in order to be effective may not be good candidates for systemic administration and may increase the likelihood of off‐target and/or cytotoxic effects.^[^ ^41^ ^]^ Regardless, additional dose‐dependent screening and cytotoxicity screening should be carried out to determine the EC50 wherein the measured outcome is functional (EAP) activity without affecting viability. It is worth noting that the log spike count per well variance was high for a number of compounds, spanning up to an order of magnitude. This is likely attributable to the time course of passive diffusion and pharmacological effect. Compounds were added manually in 1:1000 boluses. We attempted to add the compounds at the same location, angle, and velocity, but, inevitably, there will be differences in the concentration the cell initially experiences as the drug diffuses. This, in combination with the time course of the pharmacological effects, causes some compounds’ period of reduced activity to straddle the initial 3 h recording. In short, we presume, based on the shape of the firing rate histograms, that some compounds with indirect mechanisms of action (e.g., MEK, MAPK) may have caused peak activity reductions after the 3 h recording was complete.

As further discussed in the following section, some compounds in this and future screenings may also affect glial function; affecting sensory function in turn. This phenomenon has been observed in vivo,^[^ ^42^, ^43^, ^44^ ^]^ wherein the “excitability” of glial cells may directly modulate sensory neuron activity, and in vitro.^[^ ^26^, ^45^ ^]^ To determine whether glial cell excitability was affected as a function of treatment, we stimulated and recorded glial oscillating calcium transients (OCaTs) as previously described.^[^ ^26^ ^]^ We observed statistically significant decreases in glia excitability after treatment with three of the fifteen compounds. While satellite glial cells are known to modulate sensory neuron activity, it is difficult, at present, to directly interpret results based on relative excitability alone. Since we are evaluating glial excitability at discrete time points, and the communication between glia and sensory neurons is bidirectional, the question of which was affected first remains. Additionally, glia in 2D culture systems does not envelope sensory neuron soma similar to the DRG, presumably reducing their capacity to buffer ion concentrations and affect paracrine signaling.^[^ ^44^, ^46^, ^47^ ^]^

### 3.1 Discussion of Cherry‐Picked “Hits” and “Misses”

Honokiol (hit): An entire review has been dedicated to the myriad mechanistic pathways modulated by Honokiol.^[^ ^48^ ^]^ Among those that are known to be pain‐relevant are CXCR4 receptors, STAT3, mTOR, EGFR, NF‐κB, GABAaR, and NMDA,^[^ ^49^ ^]^ as well as voltage‐gated sodium^[^ ^50^ ^]^ and delayed‐rectifier potassium^[^ ^51^, ^52^ ^]^ channels. A small number of antinociception studies have already been carried out in rodent inflammatory pain models, suggesting that Honokiol may serve as an effective treatment for both allodynia and hyperalgesia.^[^ ^53^ ^]^

Sorafenib and sorafenib tosylate (hits) are primarily known as potent inhibitors of Raf1 kinase, though it is also known to inhibit B‐Raf, VEGF receptors, and FGFR1.^[^ ^54^ ^]^ These anti‐tumorigenic mechanisms of action led to sorafenib being used as a primary treatment for hepatocellular carcinoma.^[^ ^55^ ^]^ However, its aggressive systemic use is associated with a number of adverse effects, including hand‐foot skin reactions and mild sensory neuropathy,^[^ ^56^, ^57^ ^]^ suggesting it may not be a good candidate for antinociception.

Nimodipine (hit) is a dihydropyridine calcium channel (i.e., L‐type channel) blocker that has a high affinity for cerebral blood vessels, making it a candidate for aneurysmal subarachnoid hemorrhage treatment.^[^ ^58^, ^59^ ^]^ Given voltage‐gated calcium channel involvement in the initiation and transmission of action potentials, nimodipine has already been evaluated in a number of neurological contexts,^[^ ^58^ ^]^ including clinical trials as a supplemental analgesic.^[^ ^59^ ^]^ While preliminary evidence suggests some advantages to nimodipine prescription, namely reducing necessary morphine dose and preventing morphine tolerance,^[^ ^59^ ^]^ its efficacy as a stand‐alone analgesic has not been demonstrated.^[^ ^60^ ^]^

Everolimus (hit) is an mTOR protein kinase blocker that is prescribed for the treatment of solid tumors but has also been evaluated for the treatment of refractory seizures associated with tuberous sclerosis complex.^[^ ^61^ ^]^ Like other mTOR kinase inhibitors, at relatively high, systemic dosage there are a number of reported adverse effects,^[^ ^62^, ^63^ ^]^ including sensory issues like pruritus,^[^ ^62^ ^]^ and has even been associated with complex regional pain syndrome diagnosis.^[^ ^64^ ^]^ To date, it has not been evaluated in the context of acute or chronic pain treatment, likely due to its action as an immunosuppressant.

Nicardipine (hit) is another dihydropryidine calcium channel blocker primarily prescribed for hypertensive crises (e.g., hypertension during pregnancy),^[^ ^65^ ^]^ but has also been shown to treat transitional Peyronie's disease.^[^ ^66^ ^]^ Additionally, like nimodipine, it has been studied in combination with opioids but may have limited antinociceptive benefits on its own.^[^ ^67^ ^]^

Selumetinib (miss) is known as a non‐ATP‐competitive inhibitor of MEK1/2 and, as such, has been FDA‐approved as a tumor therapy drug^[^ ^68^ ^]^ – first for tumors associated with neurofibromatosis. MEK is positioned upstream of ERK in the inflammatory pain pathway and may, therefore, be a good target for neuropathic/inflammatory pain treatment.^[^ ^69^ ^]^ However, the determination of selumetinib's IC50 in a number of cell lines plus review indicated that it may fall anywhere from 0.01 to 130 µM, suggesting that we may have screened at insufficient concentrations.^[^ ^70^ ^]^

Alternately, trametinib (hit), a highly specific and highly potent MEK1/2 inhibitor, has an IC50 of 2.2–174 nM.^[^ ^71^ ^]^ Trametinib has been studied as a suppressor of chemotherapy‐induced allodynia, with promising findings^[^ ^70^ ^]^ in mice.

Alternatively, studies have specifically identified the IC50 for lamotrigine and phenytoin (hit), which lie between 70 and 140 µM. Additionally, both have been used in preclinical studies for the reduction of chronic pain symptoms.^[^ ^72^, ^73^ ^]^ Phenytoin's mechanism of action relies on the voltage‐dependent block of voltage‐gated sodium and calcium channels.

Capecitabine (hit), an anti‐tumor medication that acts via RNA‐synthesis inhibition,^[^ ^74^ ^]^ is actually known to cause heart and abdominal pain,^[^ ^75^ ^]^ though through indirect adverse action on liver function. Its administration is often coupled with diltiazem, a calcium channel blocker, to relieve its associated painful side effects.

Levetiracetam (miss), an FDA‐approved epileptic drug, is an N‐type voltage‐gated calcium channel blocker. It also has not shown any ability to bind to sodium receptors or other subtypes of calcium receptors.^[^ ^76^, ^77^ ^]^ Although they are N‐type calcium channel blockers, they are not very effective at blocking calcium current within cells. Using primary hippocampal neurons, LEV did not prove to be very effective as the IC50 of the compound is 14.7 µM with a max percent current inhibition of 18% at 200 µM.^[^ ^77^ ^]^ Further behavioral models involving mice support this evidence. Testing mice's paw withdrawal latency with a heat plate and von Frey filaments, there was little difference between the results of the control and the mice injected with LEV.^[^ ^76^ ^]^ However, there was evidence that LEV was capable of inhibiting anesthesia‐induced hyperalgesia in the same study. There was a clear decrease in nociceptive threshold from mice who took LEV before the anesthetic compared to mice who did not take LEV,^[^ ^77^ ^]^ suggesting LEV might have a role as an anti‐hyperalgesic, preventing sensitization from occurring in the cell.

Zonisamide (miss) is an FDA‐approved anticonvulsant used as a treatment for epilepsy and is known for its ability to reduce bursting by increasing the hyperpolarizing stage during action potentials in sodium channels.^[^ ^78^ ^]^ However, it is not yet specified which type of Na+ channels are targeted by Zoni. The compound also partially inhibits T‐type calcium channels. Using a human neuroblastoma cell line, 38.3% of the inward calcium current (I_Ca_) was inhibited by 50 µM of Zoni, while 100 µM of Zoni inhibited 60% of I_Ca_. Specifically, in terms of Cav 3.2, which is a known calcium channel that may have a role in sensitization of thermal stimuli, only inhibits 15.4% I_Ca_ of the channel with 50 µM of Zoni.^[^ ^78^ ^]^ However, in a behavioral mouse model testing thermal hyperalgesia and mechanical allodynia, the mice exhibited a dose‐dependent reduction in thermal hyperalgesia and this effect lasted for more than 3 days. However, with mechanical allodynia, there was an inconsistent effect across different dose ranges.^[^ ^78^ ^]^ Zoni may still have a role in treating nociception, but it requires a large amount of the compound for it to have any effective impact. Furthermore, it will not be possible to know how effective that role is until their mechanism regarding sodium channels, and which subtypes they affect, are understood. Interestingly, the only publication relating specific channel activities involves Na_V_1.6,^[^ ^79^ ^]^ a voltage‐gated sodium channel expressed primarily by microglia. This “miss” highlights the necessity to screen for phenotypic action on both sensory neurons and glial cells.

Lamotrigine (miss) is another FDA‐approved anticonvulsant used as a treatment for epilepsy. It primarily targets sodium channels and specifically inhibits channels by competitively blocking a radioligand, [3H]batrachotoxinin‐A‐20‐α‐benzoate (BTX), which prevents the channel from inactivating during depolarization allowing a massive amount of sodium ions to pass through, in order to reduce bursting during an epileptic attack. Using mouse spinal cord cultured neurons, for Lamo to have an apparent effect in blocking BTX, 114 µM was required. The highest concentration, 250 µM, could block 65% of BTX binding.^[^ ^80^ ^]^ The drug is also able to inhibit Na_V_1.6 which is known to have a role in neuropathic pain. Using a cell line of HEK293 cell cultures, the cells required an EC50 of 217.2 µM to influence the channel. Despite this, human‐based clinical trials have demonstrated that there was very limited evidence that Lamo was effective in treating conditions that involved neuropathic pain, such as HIV‐related neuropathy, trigeminal neuralgia, or diabetic neuropathy.^[^ ^81^ ^]^ Lamo as a potential analgesic may not be possible in this as the required concentrations are too high to have any analgesic effect, limiting their role as an anticonvulsant only.

Rifampin (hit), also known as rifampicin, is an FDA‐approved drug used to treat infections such as tuberculosis, leprosy, and *Neisseria meningitides*.^[^ ^82^ ^]^ It is known to induce mycobacterial efflux pump inhibition.^[^ ^83^ ^]^ It behaves by producing an inhibitory activity and mechanism on tyrosinase with an IC50 of 90 ± 0.6 µM. Tyrosinase is a multi‐purpose enzyme: an oxidase mainly found in fungi, plant, and animal kingdoms. Additionally, rifampin has shown inhibitory activity against amyloid Beta protein aggregation and Taq RNAP (RNA Polymerase).^[^ ^83^ ^]^

VX‐745 or neflamapimod (hit), is an ATP‐competitive p38 inhibitor.^[^ ^84^ ^]^ Specifically, it has been shown to inhibit joint degeneracy within an osteoarthritis rat model; with an IC50 of ≈50 nM.^[^ ^85^ ^]^ Even though VX‐745 has a binding affinity to potassium and calcium ion channels, it does not exhibit a significant binding affinity in comparison to other drugs.^[^ ^86^ ^]^

## 4 Conclusion

Here, we report the development of a moderate‐throughput phenotypic assay for screening potential analgesics to address inflammatory pain. To the best of our knowledge, this is the first hiPSC‐based sensory co‐culture on MEAs that has been evaluated using both known agonists/antagonists as well as off‐label FDA‐approved compounds targeting pathways known to be involved in chronic pain development. Our results indicate that this approach represents an “excellent” assay based on assay quality metrics and represents a model system for drug “hit” or lead detection. Importantly, our culture protocols result in a model that exhibits stable phenotypic activity on the order of days to weeks, making it conducive to repetition and/or long‐term screening as well as useful in the development of other chronic nociception modes.

## 5 Experimental Section

### 48‐Well Microelectrode Array Preparation and hiPSC Seeding/Culture

hiPSC sensory co‐cultures were carried out as previously described,^[^ ^27^ ^]^ with minor changes regarding cell types. 48‐well MEAs from Axion Biosystems (Atalanta, GA) were first pre‐treated overnight with 3 to 5 µl of 0.1% polyethyleneimine (PEI, Sigma‐Aldrich, St. Louis, MO), washed twice using DI water, allowed to dry, and then coated with 3 to 5 µl of 20 µg ml^−1^ laminin (Sigma‐Aldrich, St. Louis, MO) for at least 1 h. Laminin was aspirated immediately prior to seeding and substrates were not allowed to dry completely. hiPSC glia (BX‐0600, BrainXell, Madison, WI) and hiPSC SN (Anatomic, Minneapolis, MN) were thawed rapidly, separately diluted in 37 °C DMEM (ThermoFisher Scientific, Waltham, MA), centrifuged at 300× g for 5 min, and reconstituted in supplemented SN medium (Anatomic's Senso‐MM without Inhibitors plus 1:1000 BrainXell astrocyte supplement). Following a viable cell count, cell solutions were mixed and co‐cultures were seeded simultaneously at a 2:3 astrocyte:sensory neuron ratio in a 5 µl droplet placed at the center of each MEA well. After cells were visually confirmed to be attached, the wells were gently flooded with 200 µl of fresh, pre‐warmed supplemented SN medium (described above) and cultures were housed in a 37 °C, 10% CO_2_ incubator at ≈90% humidity. 50% medium exchanges were performed every alternate day for the duration of the culture.

### Spontaneous and Temperature‐Evoked EAP Recordings

Multi‐well MEA recordings were carried out as previously described.^[^ ^27^, ^31^ ^]^ Briefly, using an Axion Maestro multi‐well MEA recording system (Axion Biosystems, Atlanta, GA), an adaptive ±5.5 σ threshold was applied to 1‐pole Butterworth band‐pass filtered data (12.5 kHz sampling rate, 250–3000 Hz band‐pass). Threshold‐crossing time stamps and associated waveform data were recorded for all 48 wells (768 electrodes) simultaneously. AEY% was defined as the cumulative percentage of electrodes across a plate or treatment group that exhibited ≥ 1 threshold crossing (presumptive EAP) per min. MFR was calculated as the number of EAP/s exhibited by active electrodes (excluding those that were “inactive” as defined above). Bursts and network bursts were defined using presets in Axion's NeurMetric Tool software as previously described.^[^ ^87^ ^]^ Modified Z’ metrics, first described in^[^ ^40^, ^88^ ^]^ as phenotypic assay quality metrics, were calculated as previously described.^[^ ^25^ ^]^ Temperature ramps were applied during live sensory co‐culture recordings by adjusting the stage‐plate temperature from 37 to 42 °C following a 5‐min baseline recording period. For each degree increase, as reported in the Axion Maestro AxIS software, was manually time‐stamped in the recording spike file and the recording persisted for 2 min following 42 °C being reached. The MEA plate was subsequently removed and placed back in the CO_2_ incubator. Temperature responsiveness was defined as the maximum spike count exhibited in a 30 s bin following initiation of the temperature ramp being ≥ 2× the maximum spike count exhibited in a 30 s bin during the baseline period (at 37 °C).

### OCaTs Stimulation and Recording

OCaTs were stimulated and recorded as previously described.^[^ ^26^ ^]^ Briefly, 10 repetitions of charge‐balanced biphasic voltage‐controlled waveforms (800 mV, 600 µs ph^−1^) were applied every 4 s during simultaneous recording of broadband data (12.5 kHz sampling rate, 0.1–5000 Hz band‐pass). Post‐processing and OCa^2+^Ts calculation consisted of 100 Hz cut‐off high‐pass filtering, peri‐event spectrogram analysis of both baseline pseudo‐trials and stimulation trials in Neuroexplorer (Nex Technologies, Colorado Springs, CO, USA), and subsequent calculation of total power over baseline (TPoB) with a custom MATLAB script as detailed in refs. [26, 45].

### Cytokine and Compound Screening

FDA‐approved compounds were purchased from SelleckChem (Selleck Chemicals LLC, Houston, TX, USA) as 100 µl volumes of 10 mM solutions in dimethylsulfoxide (DMSO). Compounds were added as low‐volume (1:1000 volume‐to‐volume ratio), high‐concentration boluses, resulting in a working concentration of 10 µM. Vehicle treatments were, likewise, 1:1000 volume‐to‐volume additions of DMSO (0.1% working concentration). Additions were made manually in each well via micropipette, directed away from the center of the MEA, allowing working concentrations to be reached by passive diffusion.

### Statistical Analysis

Inactive electrodes (< 1 spike per min) and inactive wells (< 4 active electrodes) were excluded from further analysis. All statistical analysis and data visualization were carried out in OriginPro (OriginLab Corporation, Northampton, MA). For MFR stability assessment and TPoB calculation, a one‐way ANOVA (with Tukey post‐hoc test) or KWANOVA (with Dunn's post‐hoc test) was conducted based on Shapiro–Wilk normality test results. AEY stability was calculated using the two‐sample test of proportion. In all cases, *p* < 0.05 was considered statistically significant. A hit was defined as the mean log spike count per well greater or less than 3× the median absolute deviation (MAD).

## Conflict of Interest

The authors declare no conflict of interest.

## Data Availability

The data that support the findings of this study are available from the corresponding author upon reasonable request.
